# A comparison of raw citrulline and citrulline peptide for increasing exercise-induced vasodilation and blood flow

**DOI:** 10.1186/1550-2783-12-S1-P18

**Published:** 2015-09-21

**Authors:** Jordan M Joy, Roxanne M Vogel, Paul H Falcone, Matt M Mosman, Aaron C Tribby, Chad M Hughes, Jonathan D Griffin, Schyler B Tabor, Dylan J LeFever, Stephen B McChaughey, Michael P Kim, Jordan R Moon

**Affiliations:** 1 MusclePharm Sports Science Institute, Denver, CO, USA; 2 Department of Human Performance, Concordia University Chicago, River Forest, IL, USA; 3 Department of Movement Science, Grand Valley State University, Allendale, MI, USA; 4 Department of Biomedical Engineering, Widener University, Chester, PA, USA; 5 The Hospitality College, Johnson and Wales University, Denver, CO, USA; 6 Department of Human Performance and Sport, Metropolitan State University, Denver, CO, USA; 7 Department of Sports Exercise Science, United States Sports Academy, Daphne, AL, USA

## Background

One goal of supplementation has been to increase blood flow to skeletal muscle during exercise. Raw L-citrulline (RC) has often been used for its vasodilatory effects, and recently, RC has been bound to a whey peptide (CP) to increase bioavailability. The purpose of the present study was to determine the acute hemodynamic effects of RC, CP, and placebo (PLA) following resistance exercise in healthy men when administered at a common, commercial dose.

## Methods

In a double-blind, crossover, placebo-controlled design, 11 recreationally-active males (28.2 ± 5.0y, 182.4 ± 5.7cm, 87.1 ± 10.3kg) ingested either 1.87 g of RC, 3.67 g of CP (citrulline content 1.87 g), or a flavor-matched, visually identical placebo (PLA) and performed 3 sets of 15 arm curls at 30 and 120 minutes post-supplementation. Brachial artery vessel diameter (VD) and blood flow volume (BFV) were measured via Doppler ultrasound at 0, 3, and 6 minutes post-exercise, corresponding to 30 (30P), 33 (33P), 36 (36P), 120 (120P), 123 (123P), and 126 (126P) minutes post-supplementation. Measurements were compared with both resting baseline (no treatment, no exercise) and active control (no treatment, exercise) values. Raw data were analyzed for all group, time, and group × time interactions using 2-way repeated-measures ANOVA. Delta values were analyzed using dependent T-tests. Alpha was predetermined at p < 0.05.

## Results

A significant (p < 0.05) group × time interaction was present for VD, which increased in CP versus PLA from resting baseline to 30P (CP: 0.58 ± 0.05; PLA: 0.55 ± 0.06cm) and 33P (CP: 0.57 ± 0.05; PLA: 0.54 ± 0.05cm). VD also significantly (p < 0.05) increased in CP versus PLA from active baseline to 30P, 33P, and 120P (CP: 0.58 ± 0.05; PLA: 0.55 ± 0.05cm). Moreover, CP significantly (p < 0.05) increased VD versus RC at 30P (RC: 0.56 ± 0.06cm), 33P (RC: 0.55 ± 0.06cm), and 36P (CP: 0.55 ± 0.05; RC: 0.53 ± 0.06cm) compared to active baselines. A significant (p < 0.05) group × time interaction existed for BFV, which increased in CP versus PLA from active baseline to 30P (CP: 686.3 ± 214.7; PLA: 554.8 ± 124.2mL/min). Additionally, significantly greater delta values were observed for VD when comparing CP and PLA at 30P, 33P, 36P, and 120P and for BFV at 30P versus active ([CP VD 30P: +0.06 ± 0.03cm; 33P: +0.04 ± 0.02; 36P: +0.03 ± 0.03cm], [PLA VD 30P: +0.02 ± 0.02; 33P: +0.02 ± 0.01; 36P: +0.01 ± 0.03cm], [CP BFV: +198.0 ± 179.6; PLA BFV: +48.2 ± 104.1mL/min]) and resting ([CP VD 30P: +0.10 ± 0.03cm; 33P: +0.08 ± 0.03; 36P: +0.07 ± 0.03cm], [PLA VD 30P: +0.06 ± 0.03; 33P: +0.06 ± 0.02; 36P: +0.05 ± 0.02cm], [CP BFV: +608.9 ± 179.6; PLA BFV: +477.4 ± 130.0mL/min]) baselines. VD and BFV delta values were significantly (p < 0.05) greater for CP than RC at 30P, and VD changes remained greater at 33P and 36P versus both active ([RC VD 30P: +0.03 ± 0.02; 33P: +0.02 ± 0.02; 36P:+ 0.01 ± 0.02cm], [RC BFV: +99.5 ± 152.2mL/min]) and resting ([RC VD 30P: +0.07 ± 0.04; 33P: +0.06 ± 0.04; 36P: +0.04 ± 0.04cm], [RC BFV: +510.5 ± 157.7mL/min]) baselines.

## Conclusions

Citrulline peptide can significantly increase vasodilation and the volume of blood flow compared to raw citrulline and placebo. Citrulline peptide may be a preferred choice over raw citrulline for athletes seeking enhanced vasodilation or blood flow.

